# Robot-assisted resection of benign splenic tumors in children

**DOI:** 10.1007/s00423-023-03208-5

**Published:** 2023-12-26

**Authors:** Duote Cai, Yan Ying, Jiani Fan, Yi Jin, Zongwei Huang, Yuebin Zhang, Shuhao Zhang, Qingjiang Chen, Zhigang Gao

**Affiliations:** 1 Department of General Surgery, Children’s Hospital, Zhejiang University School of Medicine, National Clinical Research Center for Child Health, Zhejiang Province, Hangzhou, 310051 China; 2 Department of Nephrology, Taizhou Hospital of Zhejiang Province Affiliated to Wenzhou Medical University, Zhejiang Province, Taizhou, 318054 China

**Keywords:** Robot-assisted, Benign splenic tumor, Surgery, Pediatric

## Abstract

**Purpose:**

Robotic surgery is becoming increasingly widely used in the field of pediatric surgery. The present study aimed to evaluate the safety and feasibility of robot-assisted resection of benign pediatric splenic tumors and to discuss the technical points.

**Methods:**

A total of 32 patients who were diagnosed with benign splenic tumors and underwent minimally invasive surgery from January 2017 to September 2023 were included in the study. The clinical data including demographic criteria, operative details, and postoperative outcomes were analyzed retrospectively.

**Results:**

Thirteen patients underwent robot-assisted surgery, and 19 patients underwent laparoscopic surgery. The median operation time was 150 min, with an interquartile range (IQR) of 120 to 200 min for the robot-assisted group and 140 min with an IQR of 105 to 180 min in the laparoscopic group (*P* = 0.318). Despite four cases in the laparoscopic group (21%) being converted to laparotomy because of intraoperative bleeding, compared with none in the robot-assisted group, there was no significant difference between two groups (*P* = 0.128). The intraoperative volume of blood loss was significantly less (*P* = 0.041), and the hospitalization expense was significantly higher (*P* = 0.000) in the robot-assisted group than for the laparoscopic group. There was no significant difference in patients’ age, tumor size, postoperative feeding time, and the postoperative hospitalization time between two groups (*P* > 0.05).

**Conclusion:**

Robot-assisted benign splenic tumor resection was safe and feasible, and it reduced surgical trauma for the pediatric patient.

## Introduction

Primary splenic tumors are rare in children and mostly benign, consisting mainly of splenic cysts and hemangiomas [1]. Abdominal computer tomography (CT) and magnetic resonance (MRI) can assist diagnosis and help to decide if surgical resection is possible for patients with symptoms or gradual enlargement of the tumor [2].

Laparoscopic surgery has become the standard procedure for the surgical treatment of splenic disorders [3] with the development of the Da Vinci robotic surgery system facilitating a minimally invasive procedure. It also offers improved three-dimensional visualization, enhanced refined movements, camera stability, and additional degrees of freedom, providing more accurate manipulation than traditional laparoscopic surgery [4].

The aim of the present study was to summarize the authors’ experience of robot-assisted excision of benign pediatric splenic tumors, discuss the technical issues, and compare the surgical outcomes of robot-assisted and laparoscopic surgery.

## Materials and methods

### Patients and clinical data

A total of 32 patients who were diagnosed with benign splenic tumors and underwent surgical resection from January 2017 to September 2023 at the Children’s Hospital, Zhejiang University School of Medicine, were studied retrospectively, after obtaining Institute Ethics Committee approval (No. 2022-IRB-016).

Whether the patient underwent robot-assisted or conventional laparoscopic surgery was decided by the surgeon and the patient’s parents’ preference. Patients with malignant splenic tumors, managed by intervention methods such as exploratory laparotomy or those with previous abdominal surgery history, were excluded. Data collection included demographic characteristics, spleen size, operative time, intraoperative complications including bleeding volume and conversion to open surgery, postoperative hospital length of stay, and histological results.

### Surgical procedures

#### Robot-assisted surgery

**Fig. 1:**
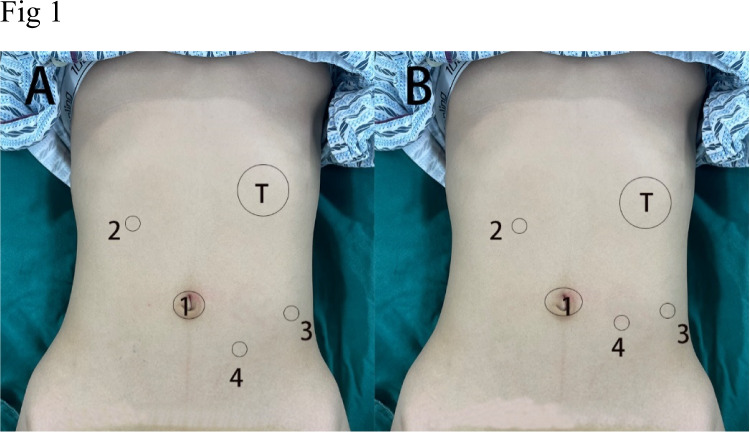
**A** Robot-assisted surgery: No. 1, 2, and 3 are the position of Da Vinci trocars; no. 4 is the position of auxiliary hole for placing the laparoscopic trocar. **B** Laparoscopic surgery: No. 1, 2, 3, and 4 are the position of laparoscopic trocars

The patient was placed in the right semi-decubitus and slightly reversed Trendelenburg position under general anesthesia to facilitate the exposure of the surgical field. After pneumoperitoneum was established, three 8-mm Da Vinci trocars and a 5-mm laparoscopic trocar were placed at the umbilical region, right upper abdomen, and left abdomen, respectively, as shown in Fig. 1A. The upper abdominal operation mode of the machine was set, the mechanical arm connected, and the operating devices installed under endoscopic monitoring.

**Fig. 2:**
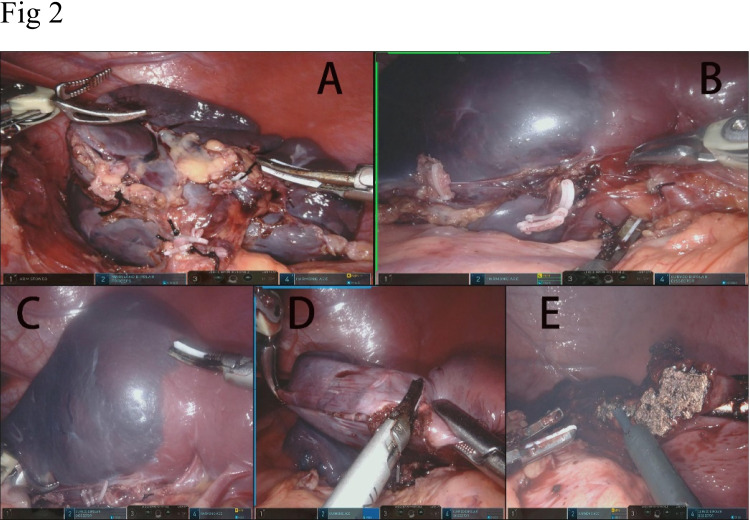
**A** For total splenectomy, the splenic artery and vein were ligated with silk thread and Hem-o-lok. **B** For partial splenectomy, the diseased splenic lobar vessels were selectively ligated by using Hem-o-lok and silk thread. **C** A clear demarcation of ischemic zone on the spleen surface. **D** The harmonic scalpel was used to resect the splenic parenchyma in the ischemic zone. **E** Hemostasis on the transection surface of the splenic remnant was completed using electrocoagulation

For total splenectomy, the harmonic scalpel was used to dissect the splenocolic, splenorenal, and splenogastric ligaments including the short gastric arteries. After all the attachments and ligaments were entirely dissected, the splenic pedicle was exposed; the splenic artery and vein were ligated with silk thread and Hem-o-lok® (Hangzhou, China) near the splenic hilum while being careful not to damage the distal pancreas, as seen in Fig. 2A.

For partial splenectomy, the perisplenic ligaments were released at the resection pole of the spleen using the harmonic scalpel. After careful dissection and exposure of the splenic hilum, the diseased splenic lobar vessels were selectively ligated by using Hem-o-lok and silk thread, seen in Fig. 2B, to expose a clear demarcation of ischemic zone in Fig. 2C. The harmonic scalpel was used to resect the splenic parenchyma in the ischemic zone in Fig. 2D. Hemostasis on the transection surface of the splenic remnant was completed using electrocoagulation, seen in Fig. 2E.

The spleen was placed in the retrieval bag, morcellated with scissors, and retracted through an enlarged umbilical incision. A drain was placed in the splenic fossa of all patients.

#### Laparoscopic surgery

Four ports were used to perform the surgery. The patient was placed in the right semi-decubitus and slightly reversed Trendelenburg position under general anesthesia. After pneumoperitoneum was established, one 8-mm and three 5-mm laparoscopic trocars were placed at the umbilical region, right upper abdomen, and left abdomen, respectively, seen in Fig. 1B. The harmonic scalpel was used for splenic ligament dissection, and the procedure for splenectomy was as for robot-assisted surgery.

### Statistical analysis

Statistical analysis was performed with SPSS 24.0. Continuous data was reported as median and interquartile ranges (IQR), the Mann–Whitney *U* test was utilized for continuous variables, and Pearson’s chi-square or Fisher’s exact tests were used for categorical data. *P* < 0.05 was considered statistically significant.

## Results

Thirteen patients including 7 males and 6 females received robot-assisted surgery, and 19 patients made up of 12 males and 7 females received conventional laparoscopic surgery. The median age was 143 months (range, 79 to 204 months) for the robot-assisted group and 131 months (range, 92 to 174 months) for the laparoscopic group. There was no significant difference in patients’ age, tumor size, operation time, postoperative feeding time, and the postoperative hospitalization time between the two groups (*P* > 0.05). The robot-assisted group had a significantly higher hospitalization cost and less intraoperative bleeding (*P* < 0.05) compared with that of the laparoscopic group, shown in Table 1.

**Table 1:** Clinical parameters of the patients

Group	*n*	Gender (male/female)	Age (months, M (IQR))	Weight (kg, M (IQR))	Surgical time (mins, M (IQR))	Intraoperative bleeding (ml, M (IQR))	Postoperative feeding time (days, M (IQR))	Postoperative hospital stay (days, M (IQR))	Tumor size (cm, M (IQR))	Hospitalization expense (RMB, M (IQR))	Converted to laparotomy (*n*)
Robot-assisted surgery	10	5/5	144 (114.75–186.5)	29 (23.35–52.25)	140 (120–207.5)	20 (5–27.5)	3 (2.75–3.25)	11 (7.75–13.5)	6.6 (4.975–9.4)	80,187.5 (74,628.5–90,956.75)	0
Laparoscopic surgery	18	11/7	129 (108–164)	39.4 (26.125–46.625)	140 (98.75–172.5)	50 (20–112.5)	3 (2.75–4)	9.5 (7.75–13)	5.7 (4.875–10.975)	27,056.5 (23,647.25–29,413)	4
*Χ*^2^/*t*/*Z*	-	-	− 1.007	− 0.552	− 0.96	− 2.117	− 0.62	− 0.604	− 0.168	− 4.315	-
*P*	-	0.698	0.314	0.581	0.337	0.034*	0.535	0.546	0.867	0.000*	0.265

The asterisk (*) indicated that the *p*-value had statistical significance

All patients in the robot-assisted group successfully underwent the minimally invasive surgery, but four cases in the laparoscopic group were converted to laparotomy because of intraoperative bleeding, though there was no significant difference in the conversion rate of laparotomy between two groups (*P* = 0.128).

Eleven patients underwent partial splenectomy, and two patients underwent total splenectomy in the robot-assisted group. In the laparoscopic group, nine patients had partial splenectomy, and ten patients had total splenectomy, but no significant difference was observed (*P* = 0.062).

One patient developed intra-abdominal encapsulated hydrops after surgery. The absorption of hydrops was unsuccessfully treated by conservative treatment, but ultrasound-guided puncture was successful. The other patients in the two groups recovered well without any other complications.

## Discussion

Though initially adopted in adult urologic procedures, the use of robotic-assisted technique is increasing in pediatric patients [5–7]. The clinical advantages of Da Vinci surgical system include stabilization of instruments within the surgical field, mechanical advantages over traditional laparoscopy, improved ergonomics for the operating surgeon, and superior visualization including three-dimensional imaging of the operative field [8].

For splenic surgery, the important step was to ligate the blood vessels in each branch for successful surgery. With the high definition vision and higher magnification of the Da Vinci surgical system, the surgeon can better dissect and ligate the blood vessels, reducing the intraoperative blood, improving the surgical success rate, and reducing the surgical trauma for patients. In the present study, all patients in the robot-assisted group underwent surgery successfully, and the intraoperative bleeding was less than that of the laparoscopic group. Four patients in the laparoscopic group were converted to laparotomy due to intraoperative bleeding that affected the surgical field.

With improved understanding of the immune function of the spleen and the distribution of the end blood vessels in the spleen, more attention has been paid to the operation of partial splenectomy, especially for children with immature immune systems [9]. If the tumor was located near the splenic hilum, or a large tumor occupied the entire spleen, total splenectomy needed to be performed, but if the tumor was located in the upper or lower pole of the spleen, partial splenectomy could be considered. The spleen is composed of three to five segments; the segmental artery originates from the polar artery and forms the terminal vascularization outside the splenic hilum. As a result, the planes between the segments are avascular, where the transection can be performed with minimal amount of blood loss [10]. Due to the high precision of the Da Vinci surgical system, the supply vessels of different segments of the spleen we can be better distinguished, allowing more accurate ligation of the blood vessels at the location of the lesion, then dissection of the splenic parenchyma within the avascular line.

For younger patients or the patients with a large tumor, robotic surgery is a better option, because these patients have a smaller operating space in the abdominal cavity, making traditional laparoscopic surgery difficult and can easily lead to surgical failure and conversion to laparotomy [11].

The three simulated wrist movement of the mechanical arm has seven degrees of freedom, which allows the surgeon to complete the operation in a narrow space, making robotic surgery more suitable for complex procedures [12].

The main drawbacks of the Da Vinci system are the long installation time, lack of tactile feedback, and high hospitalization costs. With economic improvement and reducing equipment prices, the price problem will gradually be alleviated, and the problem of time-consuming installation is gradually being solved with machine development and improvement.

The limitations of the present study are its retrospective nature and small sample size, so a prospective larger, long-term follow-up and multi-center clinical trial would be necessary to validate the advantages of this technique.

## Conclusion

Robot-assisted resection of benign splenic tumors in children is feasible and safe, its refined operation can reduce intraoperative bleeding, decrease trauma for the patient, and increase the surgical success rate. Some problems such as the high hospitalization cost are still to be solved.

## Data Availability

We are willing to make our data and study materials available to other researchers, researchers can contact ebwk@zju.edu.cn to get these materials.
